# Removal of Sodium from Vanadium Tailings by Calcification Roasting in Reducing Atmosphere

**DOI:** 10.3390/ma16030986

**Published:** 2023-01-20

**Authors:** Chao Wang, Yufeng Guo, Shuai Wang, Feng Chen, Lingzhi Yang, Yu Zheng

**Affiliations:** School of Minerals Processing and Bioengineering, Central South University, Changsha 410083, China

**Keywords:** vanadium tailings, solid waste, sodium removal, reducing, Ca(OH)_2_, phase transformation

## Abstract

Vanadium tailings from vanadium extraction by a sodium roasting process are solid waste and cannot be used in sintering and ironmaking due to their high sodium content. In this paper, a calcification and reduction roasting process was proposed to remove sodium from vanadium tailings. The effects of Ca(OH)_2_ addition, reduction temperature, and roasting time on the sodium removal behavior and compression strength of pellets were studied. The addition of Ca(OH)_2_ and the reduction of iron oxides promoted the sodium-containing phases to transform to be simpler, which could enhance sodium removal. The sodium removal rate was up to 93.47% and the compression strength of the reduced products was 4497 N/P, and the metallized ratio of the product was higher than 70% under the optimal conditions: roasting at 1200 °C for 2 h with the Ca(OH)_2_ addition of 35%. The treated product after removing sodium can be recycled in the ironmaking process in a steel company.

## 1. Introduction

Vanadium is widely used in metallurgical and chemical industries [1,2,3]. Vanadium titanomagnetite is a typical vanadium resource that contributes to about 88% of worldwide vanadium production [4,5]. Vanadium-bearing titanomagnetite generally contains around 0.2~2.5 wt% (V_2_O_5_). In the vanadium extraction process, the vanadium in vanadium titanomagnetite is reduced into molten iron and then the molten iron is oxidized in a converter to produce vanadium slag [6]. Vanadium exists in vanadium slag in the form of spinel (FeO·V_2_O_3_). Vanadium pentoxide is produced by sodium roasting following water leaching from vanadium slag [7]. Finally, the leaching residue is also known as vanadium tailing [8].

Vanadium tailings are hazardous solid wastes with high contents of alkali metals, Cr and V. In China, about 1.2 million tons of vanadium tailings are produced each year, which caused environmental problems [9]. There are two main treatment ways for vanadium tailings: to recover valuable elements and to prepare materials. Vanadium tailings have the potential to prepare far-infrared coating and vanadium titanium black porcelain. In previous research [10], vanadium tailings were calcined and then mixed with agglutinants, antifoaming agents, and dispersing agents, and a far-infrared coating was produced. Agglutinants can improve porcelain properties and the durability of the coating at high temperatures. Antifoaming agents and dispersing agents can enhance the stability of coatings. Vanadium tailings are also an excellent photothermal conversion material and magnetic material. Vanadium titanium black porcelain with different properties is obtained by adding a certain proportion of vanadium tailings into ordinary ceramic raw materials [11,12,13]. On the other hand, vanadium tailings are rich in valuable elements such as iron, chromium, vanadium, and titanium. The vanadium in tailings can be extracted by sodium roasting–water leaching [14] or direct acid leaching [15,16,17]. Furthermore, iron can be extracted using coal-based reduction and magnetic separation [8]. The tailings after the magnetic separation are also rich in V, Ti, and Cr elements.

Moreover, due to the high content of total iron in vanadium tailings, it is economical to recycle in sintering or blast furnace smelting. It may recycle Fe, V, and other valuable metals without requiring further investments. However, vanadium tailings contain high sodium content; therefore, they cannot be directly used in sintering and blast furnace smelting due to the sodium in the sintering and blast furnace, which could cause many problems [18,19,20,21]. Some researchers have tried to remove alkali metals from the tailings by alkali leaching, but a high removal efficiency of sodium cannot be obtained even with high temperatures and high pressures [5,22]. Furthermore, our group proposed the technical route of calcium roasting and NaOH leaching to remove sodium effectively [23]. However, the addition of Ca(OH)_2_ is very high and the process is also very long. Thus, it is necessary to develop a new simple way to remove sodium from vanadium tailing.

In this study, a novel method of calcification roasting and reduction was proposed, and the sodium removal behaviors and mechanism from vanadium tailings were investigated. Moreover, the metallization rate and compressive strength of pellets were also studied. After the treatment of calcium-reduction roasting, the tailing pellets satisfied the requirements of burden for blast furnace smelting. Our study will provide a new way to recycle vanadium tailing in the ironmaking process.

## 2. Experimental

### 2.1. Materials

The vanadium tailing used in this study was taken from a vanadium extraction company in China. The reduction agent used in this work is coke. Table 1 shows the industrial analysis of the coke. The chemical composition of the vanadium tailings is shown in Table 2. The total iron is 32.32 wt%, and the majority of alkali metal in the vanadium tailings is Na_2_O. It is essential to remove Na from the vanadium tailings to satisfy the requirements of a blast furnace.

**Figure 1:**
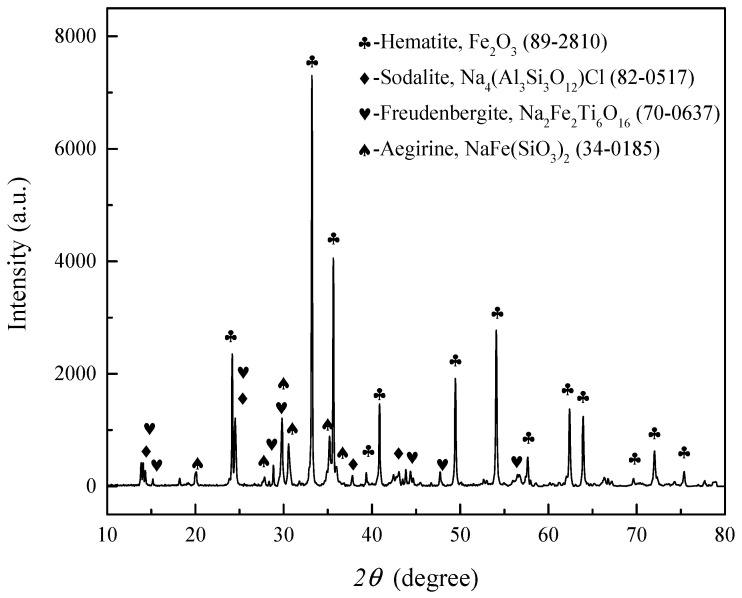
XRD pattern of the vanadium tailing.

Figure 1 is the XRD patterns of the vanadium tailings. It indicates that the main phase is hematite. Sodium primarily combines with iron, titanium, silicon, and oxygen to form complex phases. The complicated sodium-bearing phases in vanadium tailing would make the removal of sodium difficult.

### 2.2. Experimental Methods

Calcium hydroxide (Ca(OH)_2_) with analytical purity grade was used as a calcifying agent. Firstly, vanadium tailing was weighed and mixed with a designed proportion of water and Ca(OH)_2_ agent, then about 2 g sample was weighed and loaded into a mold and pressed to pellets for subsequent experiments. The pressure was controlled at about 10 N/cm^2^, and the size of the pellet was Φ10 × 10 mm.

**Figure 2:**
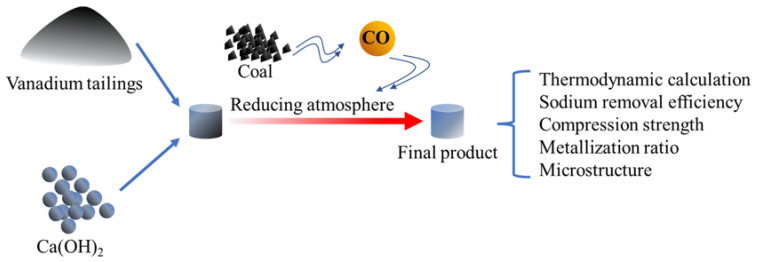
Scheme of the experiments.

The calcium reduction roasting tests were conducted in a muffle furnace with silicon-molybdenum heating elements. The operation steps of the experiments were as follows: firstly, ten pellets were placed into a 380 mL graphite crucible with about 100 g coke at the bottom, then about 200 g coke was placed and covered the pellets. The graphite crucible with the samples was placed into the muffle furnace and roasted at a designed temperature. When the reduction duration ended, the graphite crucible was cooled under Ar gas. Finally, the samples were taken from the graphite crucible and weighed, as well as performed chemical composition analysis and compressive measurements. The scheme of the experiments is shown in Figure 2.

### 2.3. Analysis and Characterization

The phase compositions of the samples in this study were determined by X-ray powder diffraction (XRD) using Cu-K radiation (50 kV, 100 mA, Bruker, Advance D8, Fällanden, Switzerland) at a scanning rate of 5°/min from 20° to 80°. The Na_2_O concentration was measured using flame atomic absorption spectroscopy (FAAS, ThermoFisher SCIENTIFIC, iCE-3500, Waltham, MA, USA). The total iron content and metal iron content in the products were measured by potassium dichromate titration. SEM and energy-dispersive spectroscopy (EDS) were used to investigate the microstructures in the samples (TESCAN, MIRA 3 LMU, Brno, Czech). WDW-QI50 pressure testing machine was used to determine the compression strength of the pellets.

### 2.4. Sodium Removal Efficiency

The sodium removal efficiency during the calcification reduction roasting can be obtained with Equation (1).

(1) $$V=(1-\frac{m_{2}\times b}{m_{1}\times a})\times 100\%$$

where V—the sodium removal efficiency;

m_1_—the mass of pellets before calcification reduction roasting;

m_2_—the mass of pellets after calcification reduction roasting;

the Na_2_O content of pellets before calcification reduction roasting;

the Na_2_O content of pellets after calcification reduction roasting.

## 3. Results and Discussion

### 3.1. Thermodynamic Analysis

**Figure 3:**
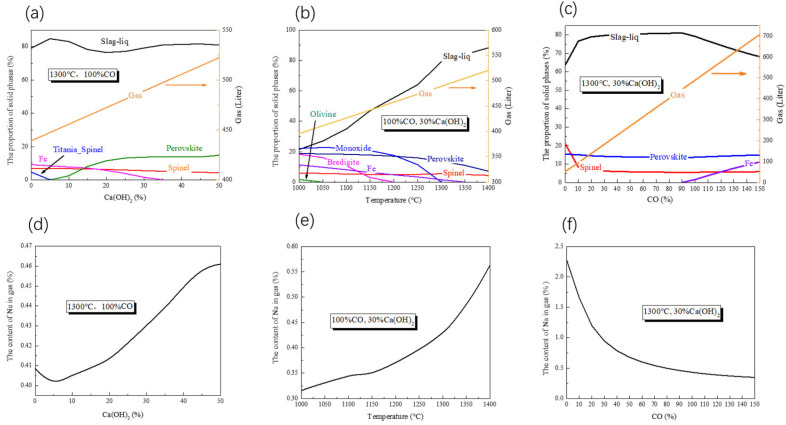
Phase composition and sodium content in gas (calculated by FactSage 8.0). ((**a**,**d**) with 1300 °C and 100% CO; (**b**,**e**) with 100% CO and 30% Ca(OH)_2_; (**c**,**f**) with 1300 °C and 30% Ca(OH)_2_).

The phase compositions of vanadium tailing during reduction roasting were calculated by FactSage 8.0 software. When Ca(OH)_2_ is increased from 0% to 50% at 1300 °C with 100% CO, the main phase is liquid phase, as shown in Figure 3a. The perovskite forms when the Ca(OH)_2_ content exceeds 5%, and its proportion increases as the added Ca(OH)_2_ increases. When the mass ratio of Ca(OH)_2_ is higher than 30%, the proportion of perovskite has slight changes. When the addition of Ca(OH)_2_ is less than 5%, there is a small amount of titania spinel phase. As the Ca(OH)_2_ addition increases, the proportions of spinel and Fe decrease. When the addition of Ca(OH)_2_ reaches 35%, the metallic iron phase disappears. The disappearance of metallic iron means that an excessive amount of calcifying agent may inhibit the reduction of ferrous oxides. Figure 3b shows the equilibrium products at different temperatures. The proportion of liquid phase increases with increasing temperature. Monoxide, perovskite, bredigite, metallic iron, and olivine have a decreasing trend with increasing temperature. The proportion of spinel is about 5% in the whole temperature range. Figure 3c depicts the effect of CO percentage on equilibrium composition. The proportion of liquid initially increases and then decreases as CO rises. The proportion of perovskite in the solid phase does not vary significantly. The proportion of spinel decreases dramatically as CO percentage increases from 0% to 10%. When the CO percentage reaches 90%, the metallic iron phase can be observed, and the proportion of metallic iron increases as the CO percentage increases. 

Figure 3a–c show that the equilibrium products include liquid, solid, and gas phases. The volume of gas is proportional to temperature, Ca(OH)_2_ concentration, and CO concentration. The proportion of sodium in the gas phases increases with increasing Ca(OH)_2_ addition and temperature. Figure 3f shows that the proportion of sodium decreases as CO concentration rises, which may be explained by more CO remaining in gas and diluting the sodium proportion.

### 3.2. Removal Behavior of Sodium

#### 3.2.1. Effect of Reduction Temperature

**Figure 4:**
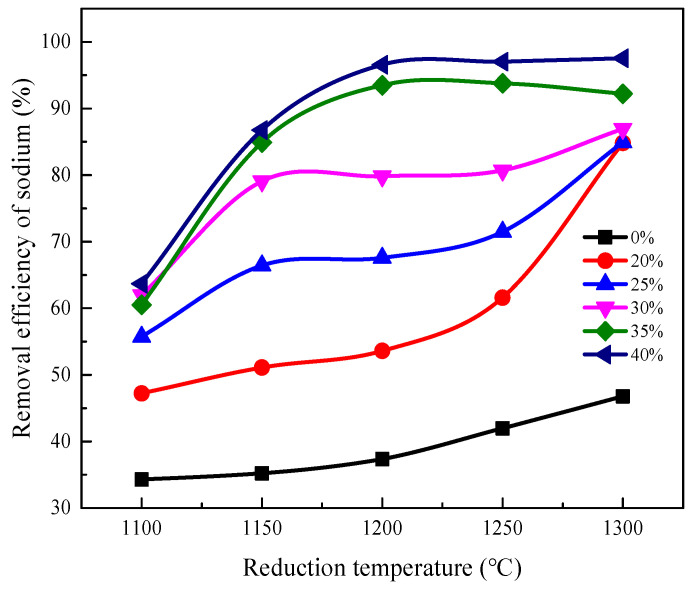
Removal efficiency of sodium at different temperatures.

The removal efficiency of sodium was investigated at different reduction temperatures with a constant duration of 2 h. The results are shown in Figure 4.

As shown in Figure 4, the removal efficiency of sodium increases with increasing roasting temperature. The removal efficiency of sodium from the samples without Ca(OH)_2_ addition is only 46.76% at 1300 °C, while it increases to 84.82% with 20% Ca(OH)_2_ addition. For the two samples with the additions of 25% and 30% Ca(OH)_2_, the sodium removal efficiency increased significantly when the reduction temperature rose from 1100 °C to 1150 °C, while at the 1150 °C to 1250 °C range, the sodium removal efficiency did not significantly increase. At the same temperature, the sodium removal efficiency of vanadium tailings with 30% Ca(OH)_2_ addition were all higher than that of 20%.

Furthermore, the sodium removal efficiency of tailings can reach more than 90% at 1200 °C when the calcium hydroxide addition increases to 35% and 40%. There is no significant increase in the sodium removal efficiency with a further increase in reduction temperature. Additionally, it can be noted that the sodium removal efficiency with 35% and 40% addition has no significant increase, which means that when the calcium hydroxide addition reaches 35%, the amount of calcifying agent is no longer a limiting factor in the sodium removal process. The added Ca(OH)_2_ can destroy the complex and stable sodium-bearing phases in vanadium tailings. The sodium-bearing phases become more simple and make it easier to volatilize. The increase in Ca(OH)_2_ addition can promote phase transformation and then improve sodium removal. The increasing temperature also promotes the volatilization of sodium compounds.

#### 3.2.2. Effect of Reduction Time

**Figure 5:**
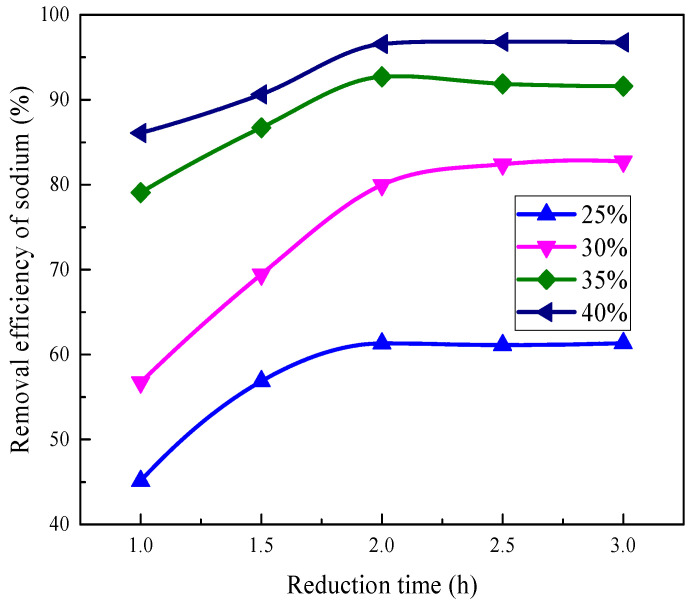
The removal efficiency of sodium from vanadium tailings for different reduction time.

The effect of reduction time on the sodium removal efficiency of the vanadium tailings with different Ca(OH)_2_ additions is shown in Figure 5. The reduction temperature was fixed at 1200 °C.

As shown in Figure 5, the removal efficiency of sodium from vanadium tailings increases with the increase in reduction time. The dramatic increase in sodium removal can be observed when the reduction time increases from 1.0 h to 2.0 h. Moreover, when the reduction time is above 2.0 h, the removal efficiencies have no significant change. It means that the volatilization of sodium may finish for 2 h. The sodium removal efficiency of the tailings for 2.0 h was 61.30%, 79.96%, 92.70%, and 96.55% when the Ca(OH)_2_ content was 25%, 30%, 35%, and 40%, respectively.

### 3.3. Reduction Behavior of Iron Oxides

**Figure 6:**
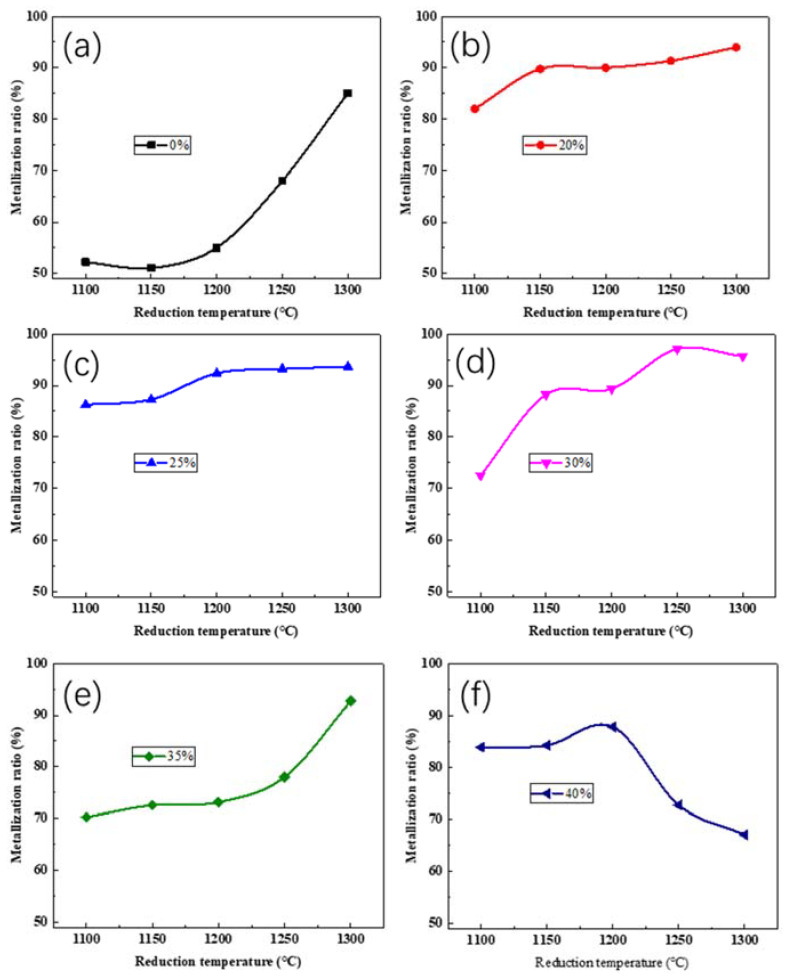
Metallization ratio of samples at different temperatures and Ca(OH)_2_ content. (**a**–**f**) represent the Ca(OH)_2_ content with 0%, 20%, 25%, 30%, 35% and 40%, respectively.

Previous research [24] showed that using the prereduced burden in blast furnace smelting can significantly reduce coke consumption. Thus, the reduction behavior of iron oxides in vanadium tailings is discussed in this part. Figure 6 depicts the metallization rates of vanadium tailings at various reduction temperatures and Ca(OH)_2_ additions. Figure 6a shows that the metallization ratio of vanadium tailings without any Ca(OH)_2_ addition increases significantly from 55.21% to 85.36% as the reduction temperature increases from 1200 °C to 1300 °C. Figure 6b,c show that the metallization ratio exceeds 80% from 1100 °C to 1300 °C with 20% and 25% Ca(OH)_2_. The metallization ratios have no large increases at 1150 °C and 1200 °C when the Ca(OH)_2_ concentration is 20% and 25%, respectively. The metallization ratio at 1100 °C is only 72.51% when the Ca(OH)_2_ content further increases to 30% (as seen in Figure 6d). The metallization ratio increases to 97.11% at 1250 °C and decreases slightly at 1300 °C. According to Figure 6e, the metallization ratio of vanadium tailings with 35% Ca(OH)_2_ is 70.26% at 1100 °C, and it increases to 91.70% when the temperature rises to 1300 °C. However, the increased reduction temperature reduces the metallization ratio when the Ca(OH)_2_ content is 40% (Figure 6f).

According to the metallization ratios with different amounts of Ca(OH)_2_, it is clear that adding the calcifying agent could improve iron oxide reduction. However, when the Ca(OH)_2_ content exceeds a certain threshold, its impact on iron oxide reduction diminishes, and iron oxide reduction is even blocked at high temperatures. Li et al. [19] demonstrated a similar law and explained that the excessive calcifying agent hampered the exchange and transfer of substances during the reduction process and prevented iron oxide reduction. The excessive addition provided barriers between the iron grains and mitigated their aggregation and growth. Furthermore, the formed liquid phases also prevented the diffusion of CO gas into the inner part of pellets.

### 3.4. Compression Strength of Reduced Pellets

**Figure 7:**
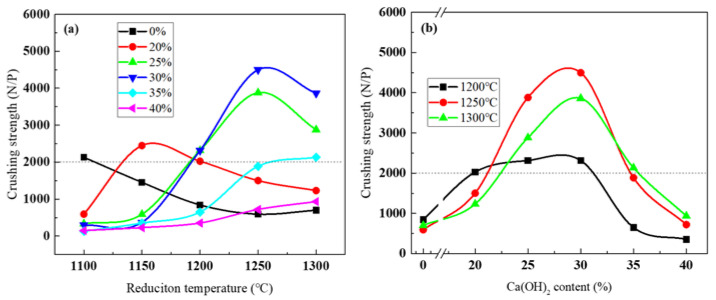
Compression strength of pellets under different conditions. The data in (**a**,**b**) are the same, with reduction temperature as the abscissa in (**a**) and Ca(OH)_2_ content as the abscissa in (**b**).

The requirements of the blast furnace burden show that the compression strength of the pellets should be higher than 2000 N/P [25,26]. As shown in Figure 7a, the compression strength of reduction products without Ca(OH)_2_ decreases as the reduction temperature increases from 1150 °C to 1300 °C. The comparison between Figure 5 and Figure 7a indicates that the increase in reduction degree is unfavorable to improving the compression strength of the pellets. This may be explained that the formation of fine metallic iron particles destroys the connection between the particles, resulting in the deterioration of the compression strength of the pellets. When the Ca(OH)_2_ addition increases to 25% and 35%, the increasing reduction temperature significantly improves the crushing strength of the pellets. When the reduction temperature reaches 1250 °C, the compression strength is 4497 N/P for the sample with 35% Ca(OH)_2_ addition.

Figure 7b shows the effect of Ca(OH)_2_ addition on the compression strength. The crushing strength of the products increases first and then decreases with the increase in calcifying agent dosage at different temperatures. The addition of less than 30% calcifying agent promotes the new bonding phase and strengthens the compression strength of the pellets. However, excessive calcium oxides remain in the products, thus weakening the coverage of the bonding phase between particles and reducing the compression strength of the pellets.

### 3.5. Microstructure of Roasted Products

**Figure 8:**
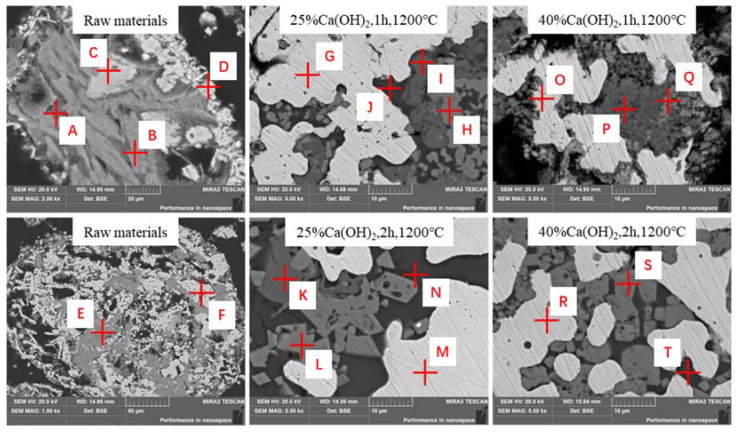
SEM-EDS results of the roasted products.

In order to analyze the mechanism of sodium removal, the roasted samples were fixed with epoxy resin, polished, and analyzed by SEM-EDS techniques. Figure 8 and Table 3 show that the vanadium tailing has complex elemental distributions and phase embedding relationships. As listed in Table 3, sodium mainly combines with silicon, iron, calcium, titanium, and aluminum. Therefore, the sodium-containing phases are very complex and stable.

The SEM images show four different phases with different grey levels (points G, H, I, and J) for the sample with the 25% Ca(OH)_2_ at 1200 °C for 1 h. Combing the EDS results in Table 3, it can be roughly determined that the main phases are metallic iron (G), spinel (H), perovskite (I), and calcium magnesium silicate (J). With the reduction time of 2 h, the microstructure and element distribution of the phases (points K, L, M, and N) are similar to the reduced products with 1 h. it can be seen that the sodium is found in the perovskite phase and calcium magnesium silicate phase. 

The SEM image of the sample with 40% Ca(OH)_2_ clearly shows three phases with different gray levels: the bright area (O), the dark area (P), and the gray area (Q). According to the EDS results, the bright phase is the metallic iron phase with minor Cr and Mn. The dark phases contain calcium silicate and perovskite phases. The gray phase is dispersive, mainly containing Ca, Si, Al, and O. After the extension of the reduction time, the proportions of calcium, aluminum, silicon, and sodium in the gray area decrease, and the proportions of chromium, manganese, titanium, and iron increase significantly. However, comparing the two regions P and T, the proportion of silicon in the dark region greatly increases, and the proportions of titanium and calcium reduce. Table 3 shows that the number of sodium-containing phases decreases with the increase in reduction time, and the content of sodium in the phases is also reduced. The phase composition and microstructures become simpler with the addition of Ca(OH)_2_ and reduction roasting. This may explain the removal of sodium with the addition of Ca(OH)_2_ and reduction roasting in this study.

### 3.6. Discussion

**Figure 9:**
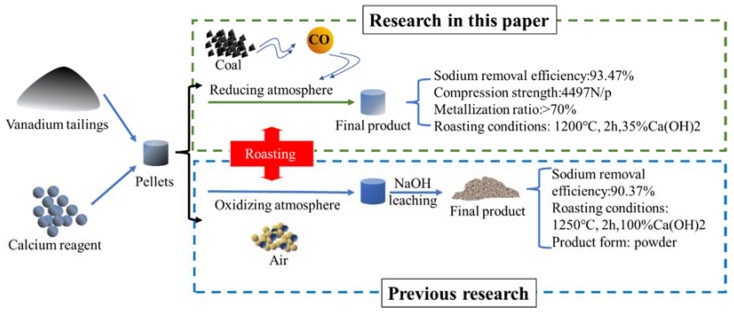
The mechanism of sodium removal in this study and previous investigations [23].

According to the EDS analysis, sodium is mainly associated with silicon, titanium, and iron in the vanadium tailings. As shown in Figure 9, at high temperatures and in a reducing atmosphere, the reduction behavior of iron promotes the separation of sodium from the iron phases, while the addition of Ca(OH)_2_ promotes the formation of the calcium-bearing silicate and perovskite and further reinforces the dissociation of sodium phases in the vanadium tailings. Under the dual action of reduction reaction and calcification reaction, more than 90% of the sodium in the vanadium tailings is separated from the original phases, and the sodium-removed products can replace part of the furnace burden for blast furnace smelting, realizing the harmless treatment of the vanadium tailings.

As illustrated in Figure 9, our previous research [23] reported sodium removal from vanadium tailings by calcium roasting in an oxidizing atmosphere followed by an alkali leaching, while the technical route of this paper is to achieve the sodium removal effect through the reduction of iron and the phase reconstruction of calcium in the reducing atmosphere. It can be seen that a higher sodium removal rate can be achieved with lower roasting temperature and a lower addition of Ca(OH)_2_. In comparison, the former has a long process, but does not need reductant, which is more carbon-friendly; the process in this paper is short, and the final product has the ideal metallization rate and compression strength, which can better match the blast furnace burden.

## 4. Conclusions

A calcium reduction roasting process was proposed to remove the sodium in vanadium tailings to make them recycled in a blast furnace. The experiments showed that increasing the reduction temperatures, reduction time, and Ca(OH)_2_ content can increase the sodium removal efficiency. The removal efficiency of sodium reached 93.47% under a reduction temperature of 1200 °C, a reduction time of 2 h, and a Ca(OH)_2_ addition of 35%. Moreover, the addition of 20% Ca(OH)_2_ was found to significantly improve the metallization ratio of the products, but excessive calcifying agents had a negative influence. When the Ca(OH)_2_ addition was more than 20%, the compression strength correlated positively with the reduction temperatures. The compression strength of the pellets increased first and then decreased as the Ca(OH)_2_ content increased. The compression strength of pellets with 30% Ca(OH)_2_ reduced at 1250 °C for 2 h reached 4497 N/P. The microstructure analysis showed that the addition of calcium could decompose the complex sodium-containing phases, and with the reduction behavior of iron in the reducing atmosphere, the sodium removal behavior in the vanadium tailings was strengthened. Compared with the early research on the removal of alkali from vanadium tailings, the process route provided in this paper has a shorter process flow and better matching with the ironmaking process.

## Figures and Tables

**Table 1:** Industrial analysis of coke (%).

FC_ad_	A_ad_	V_ad_	M_ad_
84.79	13.23	1.98	0.76

**Table 2:** Chemical composition of vanadium tailings (wt%).

Na_2_O	K_2_O	Total-Fe	SiO_2_	TiO_2_	MnO	Cr_2_O_3_	Al_2_O_3_	CaO	MgO	V_2_O_5_	Cl
4.45	0.02	32.32	13.64	11.92	6.33	4.18	2.78	1.88	1.69	1.34	0.47

**Table 3:** Chemical elemental analysis of the roasted products (by EDS).

Point	Atoms (mol%)								
	O	Ti	Ca	Cr	Mn	Fe	Al	Si	Na
A	60.52	3.75	/	3.19	7.6	24.58	/	/	/
B	41.11	1.75	9.39	/	2.82	13.24	2.93	24.97	3.79
C	38.91	10.28	/	6.15	3.52	37.92	/	/	3.22
D	53.65	3.75	/	3.19	7.6	31.22	/	/	/
E	37.49	2.76	4.80	/	1.76	10.99	1.58	26.38	10.26
F	62.74	2.91	/	4.33	4.26	20.98	/	2.84	1.80
G	/	/	/	1.14	1.62	94.87	/	/	/
H	49.88	3.10	/	12.36	11.21	/	2.72	/	/
I	66.88	13.86	14.35	/	/	/	/	/	0.87
J	63.28	/	11.55	/	2.02	1.41	2.38	14.66	2.32
K	62.83	17.03	18.41	0.67	/	0.37	/	/	/
L	54.83	2.15	/	16.42	8.09	/	2.28	/	/
M	/	/	/	/	/	100.00	/	/	/
N	63.03	/	12.18	/	1.27	/	1.87	14.96	2.05
O	/	/	/	1.82	1.05	96.40	/	/	/
P	63.89	9.57	15.38	/	/	/	1.54	5.97	1.10
Q	56.09	/	4.31	/	/	/	4.77	4.32	1.34
R	/	/	/	2.77	2.81	91.61	/	/	/
S	53.14	7.01	/	12.66	13.54	1.03	/	/	/
T	60.97	1.25	11.73	/	4.40	1.22	1.13	13.66	1.04

## Data Availability

Data is unavailable due to privacy or ethical restrictions.
